# Virtual screening by a new Clustering-based Weighted Similarity Extreme Learning Machine approach

**DOI:** 10.1371/journal.pone.0195478

**Published:** 2018-04-13

**Authors:** Kitsuchart Pasupa, Wasu Kudisthalert

**Affiliations:** Faculty of Information Technology, King Mongkut’s Institute of Technology Ladkrabang, Bangkok 10520, Thailand; Chuo University, JAPAN

## Abstract

Machine learning techniques are becoming popular in virtual screening tasks. One of the powerful machine learning algorithms is Extreme Learning Machine (ELM) which has been applied to many applications and has recently been applied to virtual screening. We propose the Weighted Similarity ELM (WS-ELM) which is based on a single layer feed-forward neural network in a conjunction of 16 different similarity coefficients as activation function in the hidden layer. It is known that the performance of conventional ELM is not robust due to random weight selection in the hidden layer. Thus, we propose a Clustering-based WS-ELM (CWS-ELM) that deterministically assigns weights by utilising clustering algorithms i.e. *k*-means clustering and support vector clustering. The experiments were conducted on one of the most challenging datasets–Maximum Unbiased Validation Dataset–which contains 17 activity classes carefully selected from PubChem. The proposed algorithms were then compared with other machine learning techniques such as support vector machine, random forest, and similarity searching. The results show that CWS-ELM in conjunction with support vector clustering yields the best performance when utilised together with Sokal/Sneath(1) coefficient. Furthermore, ECFP_6 fingerprint presents the best results in our framework compared to the other types of fingerprints, namely ECFP_4, FCFP_4, and FCFP_6.

## Introduction

Drug screening is a process of determining drug candidates that contain relevant biological targets. Recently, computers have been used to speed up the development process in order to reduce the time required to launch drugs onto the market. Moreover, it has a potential savings of millions of dollars compared to testing in vitro. Virtual screening is a set of computational techniques which aims to rank molecule structures in a database [1]. This ensures that chemists can assay molecules which have a higher probability of being active with the relevant biological target first. A conventional technique in virtual screening is called “similarity searching”. It ranks all molecules in a database on the basis of similarity or dissimilarity to a query molecule.

Machine learning techniques are becoming popular in many applications today. They also play an important role in the drug discovery process, e.g. prediction of target structures, and optimisation of hit compounds. Examples of techniques used in the drug discovery process are support vector machine (SVM) [2–4], binary discriminant analysis [2, 5], artificial neural networks [6], and decision trees [7]. Many techniques used in virtual screening have been well-documented and reviewed in the following references [8–10]. Among these techniques, SVM is one of the most powerful and popular in this area resulting in an increasing number of publications in recent decades [10].

Although SVM is a powerful algorithm, its main drawback is that it requires quadratic programming to solve the problem–at least the space complexity is quadratic. When the training dataset becomes large, its computational cost will be very intensive. In addition, SVM requires two or more user-specified parameters which directly affect the model’s performance. These parameters are required to be tuned in order to get an optimal model. Thus, the higher the number of parameters to be tuned, the more the computational cost is. In 2004, Extreme Learning Machine (ELM) was proposed by Huang et al. and made use of single hidden layer feed-forward neural network [11]. Their proposed algorithm is fast and able to obtain the optimal solution. It has proved to be competitive with SVM in performance but with a remarkable speed of training compared to SVM. Moreover, ELM requires less human intervention than SVM because the only important parameter is the number of hidden nodes [12, 13]. ELM has been applied to protein sequence classification [14–16]. To the best of our knowledge, ELM was first applied to the virtual screening task by [17] as Weighted Tanimoto ELM (WELM_JT_). The algorithm is customised for 2D binary fingerprint descriptor. WELM_JT_ replaces the activation function in neurons at the hidden layer with the Jaccard/Tanimoto (JT) similarity coefficient. Moreover, instead of randomly selecting hidden nodes with continuous distribution in the conventional ELM, WELM_JT_ randomly selects hidden nodes from the training set. Since there are many available similarity coefficients, we adopt a weighted similarity ELM (WS-ELM) algorithm which employs different similarity coefficients. This is to obtain a suitable similarity coefficient for virtual screening task with 2D fingerprint descriptor.

In addition, WS-ELM performance, like ELM, is not robust due to random weight selection. This problem should be addressed. Therefore, a deterministic assignment of hidden weights shall be considered to increase the robustness of the conventional ELM. We propose an approach to carefully select the weights of WS-ELM. Here, clustering techniques are employed to carefully select the represented candidates of weights. The proposed algorithm the so-called “Clustering based Weight Similarity ELM”(CWS-ELM) is performed and compared to the conventional techniques on well-designed experimental frameworks with one of the most challenging databases–Maximum Unbiased Validation Dataset–which consists of 17 activity classes.

## Methods

In this section, we explain all methods used in this work together with our proposed techniques.

### Similarity searching

Similarity searching is a technique to find compounds in a database which are structurally similar to a query compound. It compares the query against every single compound in the database and returns a database ranked by similarity score. Its rationale is that the more similar the structures of the molecules are, the higher the chance of them having the same properties. The degree of similarity can be calculated by similarity coefficient. Many coefficients have been introduced and re-introduced as they are in very common use in many applications [5, 18, 19].

In this paper, we investigate 16 coefficients selected from [5, 20, 21] as shown in Table 1. Some coefficients are excluded, e.g. Dice. Dice is monotonic to Jaccard/Tanimoto, therefore they give identical rankings. The similarity *s*(*x*_*i*_, *x*_*j*_) and dissimilarity *d*(*x*_*i*_, *x*_*j*_) of two molecules are usually calculated from four different quantities: (i) *a*: The number of bits set in common to both molecule *i* and *j*, (ii) *b*: The number of bits set in molecule *i* and unset in molecule *j*, (iii) *c*: The number of bits set in molecule *j* and unset in molecule *i*, and (iv) *d*: The number of bits unset in common to both molecule *i* and *j*. A combination of these four quantities (*a* + *b* + *c* + *d*) is equivalent to the number of bits *m* belonging to molecules *i* and *j*. The coefficients are divided into three main groups as follows:

- Association coefficient is based upon the inner product operation. Most of the ranges are [0, 1] which indicates no similarity and complete similarity.
- Correlation coefficients measure the degree of correlation between the molecules.
- Distance coefficients quantify the degree of difference between two objects. The more similar two objects are, the smaller the distance value is. Distance function can be converted to similarity function by *d*(***x***_*i*_, ***x***_*j*_) = 1 − *s*(***x***_*i*_, ***x***_*j*_).

**Table 1:** Formulas for similarity/dissimilarity coefficients for binary-valued vectors.

ID	Common Name	Formula	Range
C01	Baroni-Urbani/Buser	$s\left(x_{i},x_{j}\right)=\frac{\sqrt{ad}+a}{\sqrt{ad}+a+b+c}$	0 to 1
C02	Hamman	$s\left(x_{i},x_{j}\right)=\frac{a+d-b-c}{m}$	-1 to 1
C03	Jaccard/Tanimoto	$s\left(x_{i},x_{j}\right)=\frac{a}{a+b+c}$	0 to 1
C04	Kulczyński	$s\left(x_{i},x_{j}\right)=\frac{1}{2}\left(\frac{a}{a+b}+\frac{a}{a+c}\right)$	0 to 1
C05	Cosine/Ochiai	$s\left(x_{i},x_{j}\right)=\frac{a}{\left(a+b)(a+c\right)}$	0 to 1
C06	Roger/Tanimoto	$s\left(x_{i},x_{j}\right)=\frac{a+d}{b+c+m}$	0 to 1
C07	Russell/Rao	$s\left(x_{i},x_{j}\right)=\frac{a}{m}$	0 to 1
C08	Simple Match	$s\left(x_{i},x_{j}\right)=\frac{a+d}{m}$	0 to 1
C09	Simpson	$s\left(x_{i},x_{j}\right)=\frac{a}{\text{min}(a+b,a+c)}$	0 to 1
C10	Sokal/Sneath(1)	$s\left(x_{i},x_{j}\right)=\frac{a}{a+2b+2c}$	0 to 1
C11	Sokal/Sneath(2)	$s\left(x_{i},x_{j}\right)=\frac{2a+2d}{a+d+m}$	0 to 1
C12	Sokal/Sneath(3)	$s\left(x_{i},x_{j}\right)=\frac{ad}{\left(a+b)(a+c)(d+b)(d+c\right)}$	0 to 1
C13	McConnaughey	$s\left(x_{i},x_{j}\right)=\frac{a^{2}-bc}{\left(a+b)(a+c\right)}$	-1 to 1
C14	Pearson	$s\left(x_{i},x_{j}\right)=\frac{ad-bc}{\sqrt{\left(a+b)(a+c)(b+d)(c+d\right)}}$	-1 to 1
C15	Yule	$s\left(x_{i},x_{j}\right)=\frac{ad-bc}{ad+bc}$	-1 to 1
C16	Mean Manhattan	$d\left(x_{i},x_{j}\right)=\frac{b+c}{m}$	1 to 0

If multiple active molecules (*n*_*A*_) are available, we can calculate the similarity value between a molecule ***x***_*j*_ in the unranked database and a set of query molecules–for all ***x***_*i*_ ∈ Actives by,

$$\begin{array}{c} s_{A}\left(x_{j}\right)=\frac{1}{n_{A}}\sum_{i\in \text{Actives}}s\left(x_{i},x_{j}\right). \end{array}$$ (1)

### Extreme Learning Machine

Extreme Learning Machine (ELM) was first proposed by Huang et al. [11]. It is based on a single layer feed-forward neural network architecture. Consider the matrix of *m*-dimensional sample vectors X = [***x***_1_, ***x***_2_, …, ***x***_*n*_]^T^ and a target vector ***y*** comprising *y*_*i*_ ∈ {−1, +1}. The output of ELM can be defined as a linear sum of weights (*β*_*i*_)–connecting the hidden neurons to the output–associated with the hidden layer outputs. There are *l* nodes in the hidden layer. The hidden layer outputs use an activation function *g*(⋅) with a linear combination of input ***x*** and synaptic weights (***w***_*i*_) and bias (*b*_*i*_)–connecting the hidden neuron to the input neurons–as function input. Therefore the model can be defined as:

$$\begin{array}{c} y_{j}=\sum_{i=1}^{l}\beta_{i}g\left({w_{i}}^{\text{T}}\cdot x_{j}+b_{i}\right) \end{array}$$ (2)

where ***w***_*i*_ = [*w*_*i*1_, …, *w*_*im*_] (randomly generated). Therefore, the activity of the hidden node can be represented as

$$\text{H}=\left[\begin{array}{c} h(x_{1})\\ \vdots \\ h(x_{n}) \end{array}\right]={\left[\begin{array}{ccc} g(w_{1}{}^{\text{T}}\cdot x_{1}+b_{1}) & \ldots & g(w_{l}{}^{\text{T}}\cdot x_{1}+b_{l})\\ \vdots & \ddots & \vdots \\ g(w_{1}{}^{\text{T}}\cdot x_{n}+b_{1}) & \ldots & g(w_{l}{}^{\text{T}}\cdot x_{n}+b_{l}) \end{array}\right]}_{n\times l}.$$ (3)

The ELM aims to minimise the mean squared error,

$$\begin{array}{c} \text{min}\frac{1}{2}\sum_{i=1}^{n}\left|\right|y_{i}-{\hat{y}}_{i}{\left|\right|}_{2}^{2}. \end{array}$$ (4)

where ${\hat{y}}_{i}$ is a predicted target. Thus, Moore-Penrose pseudo-inverse is employed to achieve the optimal solution for this problem. Hence, ***β*** can be defined by,

$$\begin{array}{c} \beta ={\left(H^{T}H\right)}^{-1}H^{T}y. \end{array}$$ (5)

The prediction score can be computed from

$$\begin{array}{c} \hat{y}=H\beta . \end{array}$$ (6)

### Weighted Similarity Extreme Learning Machine

The proposed Weighted Similarity ELM (WS-ELM) consists of two functions which are (i) empirical likelihood function–mean squared error–and (ii) penalised likelihood functions–ridge penalty,

$$\begin{array}{c} \underset{\beta }{\text{min}}\frac{1}{2}{\left\|\text{H}\beta -y\right\|}_{2}^{2}+\frac{1}{C}{\left\|\beta \right\|}_{2}^{2}. \end{array}$$ (7)

The activation function *g*(⋅) in the conventional ELM is replaced by *s*(⋅, ⋅), hence, the H is represented as,

$$\text{H}={\left[\begin{array}{ccc} s(x_{1},w_{1}) & \ldots & s(x_{1},w_{l})\\ \vdots & \ddots & \vdots \\ s(x_{n},w_{1}) & \ldots & s(x_{n},w_{l}) \end{array}\right]}_{n\times l}.$$ (8)

*C* is a regularisation parameter to control the complexity of the model. ***w*** is randomly selected from the training set ***w*** ⊂ X instead of randomly selected from a continuous distribution. This is to ensure that the achieved weights are binary, sparse, and have identical dimension span.

The virtual screening task faces a dramatic imbalance between the number of active (*n*_*A*_) and inactive (*n*_*I*_) molecules. In order to deal with this imbalanced class problem, a diagonal Γ_*n*×*n*_ is defined associated with all training samples. A minority class will be given higher importance than a majority class. Thus, the likelihood function becomes

$$L(\beta )=\frac{1}{2}\Gamma {\left\|\text{H}\beta -y\right\|}_{2}^{2}+\frac{1}{C}{\left\|\beta \right\|}_{2}^{2}$$ (9)

$$=\frac{1}{2}{\left\|\sqrt{\Gamma }\text{H}\beta -\sqrt{\Gamma }y\right\|}_{2}^{2}+\frac{1}{C}{\left\|\beta \right\|}_{2}^{2}.$$ (10)

The above likelihood function can be minimised using standard ℓ_2_-regularised weighted least squares which gives the following solution

$$\begin{array}{c} \beta ={\left(\frac{\text{I}}{C}+{\text{H}}^{\text{T}}\Gamma \text{H}\right)}^{-1}{\text{H}}^{\text{T}}\Gamma y. \end{array}$$ (11)

Instead of calculating H^T^ΓH, we can calculate (***γ*** ⋅ H)^T^(***γ*** ⋅ H), where $\gamma =\text{diag}(\sqrt{\Gamma })$. This technique can speed up the computational time [17]. This leads to the solution in Eq 12.

$$\begin{array}{c} \beta ={\left(\frac{\text{I}}{C}+{\hat{\text{H}}}^{\text{T}}\hat{\text{H}}\right)}^{-1}{\hat{\text{H}}}^{\text{T}}\gamma \cdot y, \end{array}$$ (12)

where $\hat{H}=\gamma \cdot H$. *γ*_*i*_ can be defined as,

$$\begin{array}{c} \begin{array}{c} \gamma_{i}=\{\begin{array}{cc} \sqrt{\frac{\max(n_{I},n_{A})}{n_{A}}} & ;\text{if}\;y_{i}=\text{Active}\\ \sqrt{\frac{\max(n_{I},n_{A})}{n_{I}}} & ;\text{if}\;y_{i}=\text{Inactive} \end{array} \end{array} \end{array}$$ (13)

**Fig 1:**
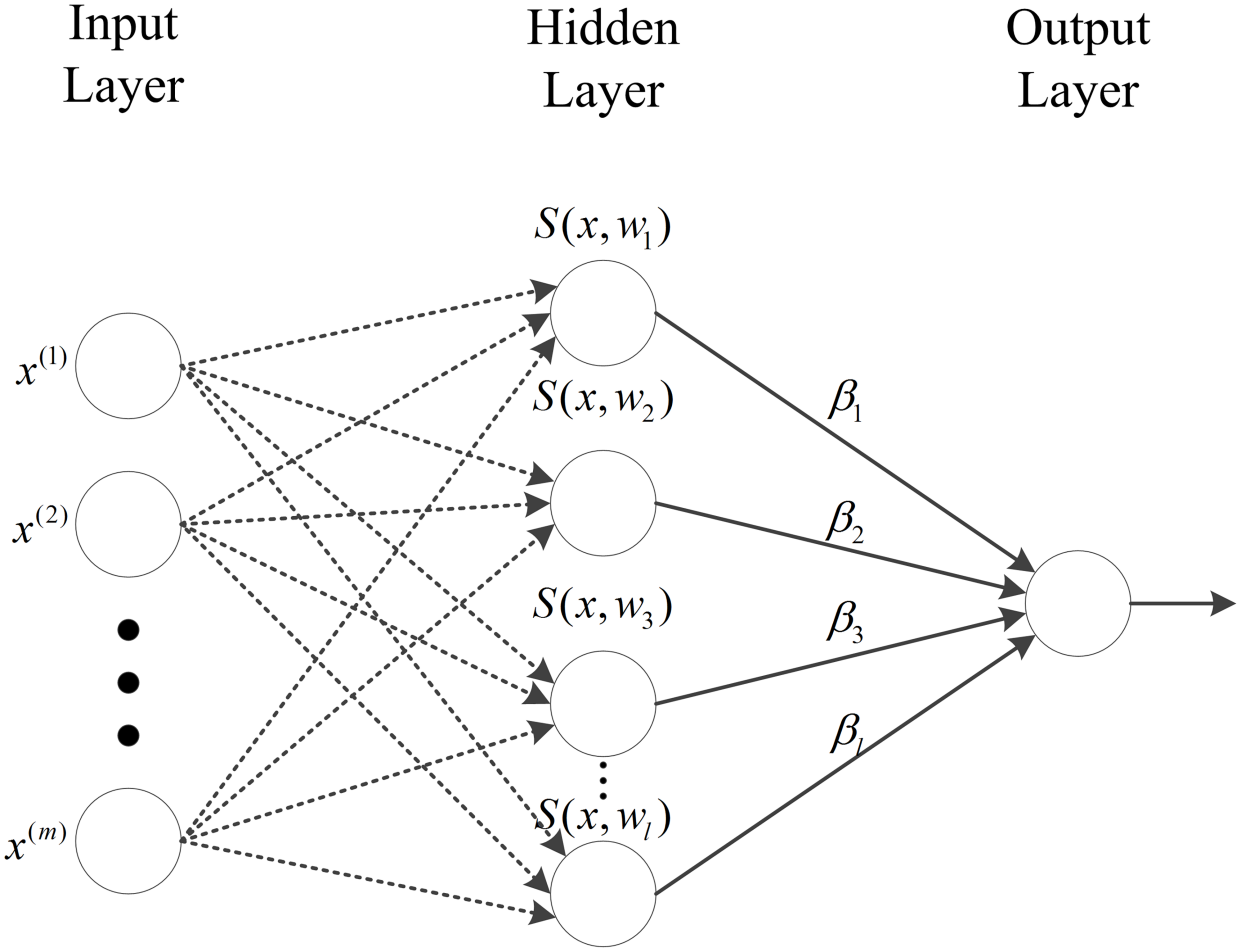
Architecture of the WS-ELM.

The architecture of WS-ELM is shown in Fig 1.

### Clustering-based Weighted Similarity Extreme Learning Machine

Due to randomness of weights between input and hidden layers, the prediction of the conventional ELM is not stable. This is applicable to the case of WS-ELM as well because a subset of samples in the training set is randomly selected to represent the weights in WS-ELM. Therefore, a deterministic assignment of hidden weights will be able to improve the performance of the conventional ELM. In order to enable the deterministic approach to this, we utilise cluster analysis methods to organise and summarise data through group prototypes. Thus, we propose a new algorithm called “Clustering-based WS-ELM”(CWS-ELM).

Clustering analysis is an unsupervised learning technique for grouping samples in the space into *k* groups. It aims to minimise the distance of samples within each cluster while maximising the distance between groups. Many clustering algorithms have been introduced and well-documented [22, 23]. In this paper, we investigate *k*-mean clustering and support vector clustering algorithms. The rationale behind this selection is the choice of representation of the data for each group. A cluster can be represented by its centroid identified by *k*-mean clustering algorithm or a set of samples bounding the cluster. Brief details of these two algorithms are explained in the following subsection. The pseudo-code for CWS-ELM is shown in Algorithm 1.

#### *k*-mean clustering

This is the conventional clustering technique which aims to minimise the Euclidean distance between the samples and the centroid in each cluster. The number of clusters (*k*) must be determined by the user. Instead of Euclidean distance, we can adopt other distance- or similarity-coefficients listed in Table 1 as well. In order to ensure that CWS-ELM will pick a binary weight, we choose a sample that is the closest to the centroid. Thus, the number of nodes used in CWS-ELM is equal to the number of centroids representing all clusters in the training data.

#### Support vector clustering

Support Vector Clustering (SVC) is inspired by a well-known algorithm, the so-called “Support Vector Machine”and is introduced by [24]. SVC employs a kernel trick to map all samples into a high dimensional feature space and obtains the smallest sphere which contains the mapped samples. The sphere can be mapped back to the original feature space and forms a set of contours which enclose samples. Samples in the same contour are hosted in the same cluster. Furthermore, any points lying on the boundary of the sphere–cluster boundary–are considered as support vectors. Moreover, embedding a soft margin in SVC can enable the sphere not to enclose all points in it. Thus the algorithm can have the ability to deal with outliers. The similarity function in Table 1 can be adopted as a kernel function similar to [5]. In CWS-ELM, the number of nodes is equivalent to the number of support vectors bounding each clusters in the training data.

**Algorithm 1** Clustering-based Weighted Similarity Extreme Learning Machine

1: **function** CWS-ELM_Train(X, ***y***, method)

2:  **switch** method **do**

3:   **case** 0                     ▹Conventional

4:    W ← Randomly select a subset of X

5:   **case** 1                   ▹*k*-mean Clustering

6:    W ← Centroid of each cluster by *k*-mean

7:   **case** 2                         ▹SVC

8:    W ← Support vector bounding each cluster by SVC

9:  *n* ← #samples

10:  *n*_*A*_ ← #positive samples

11:  *n*_*I*_ ← #negative samples

12:  **for** *i* ← 1 to *n* **do**

13:   **if** *y*_*i*_ = 1 **then**

14:    $\gamma_{i}\leftarrow \sqrt{\frac{\max(n_{I},n_{A})}{n_{A}}}$

15:   **else**

16:    $\gamma_{i}\leftarrow \sqrt{\frac{\max(n_{I},n_{A})}{n_{I}}}$

17:   **end if**

18:  **end for**

19:  $\hat{H}\leftarrow \gamma \cdot S\left(X,W\right)$

20:  $\beta \leftarrow {\left(\frac{I}{C}+\hat{H}H\right)}^{-1}\left(\hat{H}\gamma \cdot y\right)$

21:  **return** W, ***β***

22: **end funtion**

23: **function** CWS-ELM_Predict(W, ***β***, X_Test_)

24:  H ← S(X_Test_, W)

25:  $\hat{y}\leftarrow H\beta$

26:  **return t**

27: **end funtion**

## Dataset and experiment framework

### Maximum Unbiased Validation Dataset

The experiments were conducted on a well-known open to the public dataset in a virtual screening task using the so-called “Maximum Unbiased Validation”(MUV) dataset which was created by the Institute of Pharmaceutical Chemistry, Braunschweig University of Technology, Germany [25]. The dataset consists of 17 bioactivity data sets carefully selected from PubChem–an open archive of the biological activities of millions of molecules as shown in Table 2. Each set consist of 30 active compounds together with 15,000 carefully selected confirmed inactive compounds (also known as decoys). An active compound is a compound which causes a corresponding biological activity while an inactive compound does not. The active compounds in each activity are designed to be structurally heterogeneous (sometimes called diverse) with only 1.14 compounds on average of distinct scaffolds in each activity class. The scaffold is the core structure which is the main component of a molecule. Moreover, the classes are grossly imbalanced with over 99.8% belonging to the inactive group. Therefore, this dataset is one of the most challenging in virtual screening tasks.

**Table 2:** The 17 activity classes in the MUV dataset. The entries are ranked in decreasing order of average mean pairwise similarity across four fingerprints.

ID	Activity Class	AID Key	#Scaffolds	$\frac{\#\text{Active}}{\#\text{Scaffold}}$	Mean Pairwise Similarity Score				
					ECFP_4	ECFP_6	FCFP_4	FCFP_6	Average
I01	FXIa Inhibitors	846	21	1.43	0.22	0.18	0.28	0.22	0.23
I02	FXIIa Inhibitors	852	24	1.25	0.22	0.18	0.26	0.21	0.22
I03	Cathepsin G Inhibitors	832	24	1.25	0.21	0.18	0.25	0.20	0.21
I04	PKA Inhibitors	548	27	1.11	0.23	0.16	0.23	0.18	0.20
I05	Rho-Kinase 2 Inhibitors	644	27	1.11	0.19	0.16	0.24	0.19	0.20
I06	ER-*α*-coactivator-binding Potentiators	737	28	1.07	0.19	0.16	0.24	0.19	0.19
I07	M1 Receptor Allosteric Inhibitors	859	29	1.03	0.19	0.16	0.24	0.19	0.19
I08	SF1 Inhibitors	600	24	1.25	0.18	0.16	0.23	0.18	0.19
I09	S1P1 Receptor Agonists	466	28	1.07	0.18	0.15	0.23	0.18	0.18
I10	FAK Inhibitors	810	28	1.07	0.16	0.14	0.22	0.18	0.18
I11	D1 Receptor Allosteric Modulators	858	24	1.25	0.17	0.15	0.21	0.17	0.17
I12	ER-*β*-coactivator-binding Inhibitors	733	28	1.07	0.17	0.15	0.22	0.17	0.17
I13	ER-*α*-coactivator-binding Inhibitors	713	26	1.15	0.17	0.15	0.21	0.17	0.17
I14	SF1 Agonists	692	30	1.00	0.16	0.14	0.22	0.17	0.17
I15	Eph Receptor A4 Inhibitors	689	29	1.03	0.17	0.14	0.21	0.17	0.17
I16	HIV RT-RNase Inhibitors	652	27	1.11	0.15	0.13	0.22	0.17	0.17
I17	HSP 90 Inhibitors	712	27	1.11	0.16	0.14	0.20	0.16	0.16
	Average		26.53	1.14	0.18	0.15	0.23	0.18	0.19

We represent the data with two popular fingerprints generated by Pipeline Pilot software, namely: Extended Connectivity Fingerprint (ECFP), and Functional-class Fingerprint (FCFP) [26]. The reason behind the selection of these two types of fingerprints is that Gardiner et al. demonstrated that ECFP and FCFP fingerprints yielded the best two fingerprints among BCI [27], Daylight [28], ECFP, FCFP, MDL [26], and Unity [29] fingerprints in virtual screening tasks [30]. In this work, both ECFP and FCFP fingerprints utilise a circular substructure of four or six diameter bonds–represented as ECFP_4, ECFP_6, FCFP_4, and FCFP_6. All four types of fingerprints have a fixed dimension of 1,024-D.

As mentioned earlier the MUV dataset is very diverse; another widely used indicator for diversity among substructures of molecules in a database is mean pairwise similarity (MPS) score. The lower the score, the more heterogeneous an activity class is. Hence, it will be very difficult to identify/retrieve in a virtual screening task. The MPS of each compound with every other compound in the class, calculated with different fingerprints using the Jaccard/Tanimoto similarity coefficient is shown in Table 2. It can be seen that the MPS on average is only 0.19/1.00.

### Experiment settings

The dataset is divided into training and test sets. The training sets are created similarly to [30–32]. All 30 active molecules from the 17 activity classes in the MUV dataset are collected in a data pool. Then, we randomly select 170 molecules (*n*_*Tr*_) as a training set which consists of 10 active and 160 inactive molecules for each activity class under consideration. A set of the remaining samples in the data pool combined with inactive samples for each activity class under consideration constitute a test set. Active and inactive molecules are labelled as 1 and −1, respectively.

The experiments are divided into three parts as follows:

- Evaluating WS-ELM in conjunction with different types of available similarity coefficients against the baseline method–similarity searching–on four considered fingerprints in order to obtain the best similarity coefficient suitable for WS-ELM and fingerprint.
- Comparing our proposed algorithm, CWS-ELM, with ELM variants.
- Comparing the proposed algorithm with other approaches, i.e. Similarity Searching, SVM, and Random Forests (RF).

All experiments are run 10 times with different random splits on training and test sets.

In addition, hyper-parameters in each algorithm are identified by estimating generalisation error via five-fold cross-validation on the basis of the area under the Receiver Operating Characteristic Curve (AUROC) on the training set. There are many criteria for evaluation of virtual screening tasks, e.g. AUROC, Enrichment Factor (EF), Robust Initial Enhancement (RIE), Boltzmann-Enhanced Discrimination of ROC [33–36]; but we select AUROC because it is simple and a standard metric for many fields.

In WS-ELM, there are two parameters which need to be tuned: number of hidden nodes (*l*) and a regularisation parameter (*C*). The range of *l* was [1, …, *n*_*Tr*_] while the range of *C* was [10^−6^, 10^−5^, …, 10^5^, 10^6^]. For CWS-ELM, the regularisation parameter (*C*) is required to be tuned using the same range as WS-ELM. In addition to the base hyper-parameter of WS-ELM, the number of clusters (*k*) for *k*-mean-based WS-ELM (CWS-ELM_KMC_) is required to be within a range of 1 to *n*_*Tr*_, while SVC-based WS-ELM (CWS-ELM_SVC_) has another regularisation *C*_*S*_ with a range from 0.1 to 1.0 with increments of 0.1.

A model is trained with the training data with a set of optimal parameters. The model is tested on the test set and evaluated with a widely used performance measure in a virtual screening task–the average proportion of the maximum possible number of active molecules (hit rate) which is retrieved from the top 1% of the ranked database. The molecules are ranked based on the predicted score from the output layer of WS-ELM and its variants. The higher the score, the more likely the molecule is to be active.

All experiments are carried out using the Matlab environment. SVC toolbox is available to download at https://sites.google.com/site/daewonlee/research/svctoolbox and the proposed CWS-ELM can be downloaded at https://github.com/dsmlr/cwselm.

## Results and discussions

### A comparison of similarity searching and WS-ELM with the 16 similarity coefficients on four types of fingerprint

WS-ELM together with the 16 coefficients and similarity searching were evaluated on the 17 activity classes with four types of fingerprint. The experiment results are shown in Tables 3 and 4 for similarity searching and WS-ELM, respectively. Each element in these tables contains the mean hit rate, when averaged across the four fingerprints and 10 different data splits, in the top 1% of the ranked database. It is clear that Sokal/Sneath(1) could achieve the best performance followed by Jaccard/Tanimoto and Sokal/Sneath(3) coefficients in both similarity searching and WS-ELM techniques. It should be noted that Sokal/Sneath(1) is a modified version of the Jaccard/Tanimoto function which gives double weight to non-matches. The worst similarity coefficients in similarity searching and WS-ELM are Roger/Tanimoto and Yule, respectively.

**Table 3:** Maximum percentage actives retrieved in top 1% of ranked database using similarity searching technique (average across 10 runs). Bold face is the best result in each activity class.

Class	Similarity Coefficient																Mean
	C01	C02	C03	C04	C05	C06	C07	C08	C09	C10	C11	C12	C13	C14	C15	C16	
I01	23.88	8.75	31.38	28.88	29.25	8.63	19.50	8.75	21.50	**32.88**	28.75	29.50	28.88	29.25	16.25	8.75	22.17
I02	28.63	13.25	33.38	31.13	31.38	13.25	16.75	13.25	24.00	**34.50**	31.50	31.75	31.13	32.13	19.13	13.25	24.90
I03	13.37	3.00	26.63	22.13	22.25	3.00	23.88	3.00	19.38	**28.88**	21.75	22.63	22.13	21.88	4.63	3.00	16.35
I04	16.50	5.88	20.75	18.63	18.75	6.00	9.13	5.88	12.50	**22.63**	19.00	18.88	18.63	19.25	8.88	5.88	14.20
I05	15.63	9.75	20.63	19.00	19.00	9.38	6.63	9.75	14.88	**22.25**	19.38	19.38	19.00	19.13	11.13	9.75	15.29
I06	0.75	1.50	2.75	2.13	2.38	1.38	**4.00**	1.50	0.25	3.25	1.88	2.38	2.13	2.13	0.25	1.50	1.89
I07	2.50	0.00	4.75	3.75	3.63	0.00	3.38	0.00	2.38	**5.13**	3.75	3.63	3.75	3.75	1.38	0.00	2.61
I08	0.75	0.00	6.88	3.50	3.50	0.00	4.13	0.00	1.50	**8.38**	3.00	3.75	3.50	3.38	0.25	0.00	2.66
I09	0.88	0.00	3.75	2.13	2.25	0.00	3.00	0.00	1.63	**4.75**	1.88	2.13	2.13	2.00	0.38	0.00	1.68
I10	2.00	2.50	5.13	3.50	3.63	2.50	5.38	2.50	3.75	**6.25**	3.63	3.75	3.50	3.75	0.50	2.50	3.42
I11	2.75	0.50	4.00	2.50	2.50	0.38	0.75	0.50	1.38	**5.50**	2.25	2.50	2.50	2.25	2.88	0.50	2.10
I12	2.63	0.50	3.25	2.63	2.63	0.50	1.63	0.50	1.88	**4.38**	2.63	2.63	2.63	2.63	1.75	0.50	2.08
I13	2.00	1.13	4.88	3.13	3.25	1.13	3.50	1.13	3.25	**6.00**	3.00	3.25	3.13	3.13	0.63	1.13	2.73
I14	1.75	0.50	**1.88**	1.75	1.50	0.50	0.75	0.50	1.13	**1.88**	1.75	1.63	1.75	1.63	0.88	0.50	1.27
I15	4.50	0.88	6.75	5.25	5.25	0.88	1.50	0.88	2.50	**8.25**	6.00	5.50	5.25	6.00	2.75	0.88	3.94
I16	0.50	0.00	4.13	2.88	2.88	0.00	4.50	0.00	1.75	**5.88**	2.13	2.75	2.88	2.25	0.13	0.00	2.04
I17	2.13	0.00	**6.00**	4.13	4.63	0.00	3.38	0.00	2.25	**6.00**	4.13	4.63	4.13	4.25	0.88	0.00	2.91
Mean	7.13	2.83	11.00	9.24	9.33	2.80	6.58	2.83	6.82	**12.16**	9.20	9.45	9.24	9.34	4.28	2.83	7.19

**Table 4:** Maximum percentage actives retrieved in top 1% of ranked database using WS-ELM technique (average across 10 runs). Bold face is the best result in each activity class.

Class	Similarity Coefficient																Mean
	C01	C02	C03	C04	C05	C06	C07	C08	C09	C10	C11	C12	C13	C14	C15	C16	
I01	14.25	24.38	25.50	25.63	26.00	25.13	23.13	24.38	20.00	25.88	24.88	**26.75**	25.63	24.63	10.00	24.38	23.16
I02	14.00	26.50	27.13	28.63	28.13	27.50	24.00	26.50	25.25	28.00	29.13	28.25	28.63	**29.50**	9.50	26.50	25.45
I03	7.25	22.63	27.25	22.50	23.50	23.63	23.75	22.63	18.00	**27.63**	24.25	23.88	22.50	24.50	1.00	22.63	21.09
I04	8.50	20.50	**26.00**	19.63	20.88	20.00	22.13	20.50	20.50	25.25	21.13	21.25	19.63	22.88	5.63	20.50	19.68
I05	5.63	16.38	22.13	18.00	18.63	17.88	18.63	16.38	15.75	**23.00**	18.38	18.63	18.00	17.75	4.25	16.38	16.61
I06	1.75	2.50	2.25	2.00	2.13	2.88	**3.63**	2.50	2.13	2.63	1.75	2.13	2.00	1.25	2.38	2.50	2.27
I07	1.25	2.13	**3.75**	3.13	3.25	2.00	2.38	2.13	2.00	3.38	2.50	3.13	3.13	2.75	1.88	2.13	2.55
I08	2.75	3.63	**7.63**	4.88	5.50	4.25	5.63	3.63	5.50	7.25	5.38	6.13	4.88	5.88	0.75	3.63	4.83
I09	1.88	3.25	3.75	2.00	1.75	3.25	2.63	3.25	2.38	**4.25**	2.50	1.75	2.00	2.63	1.00	3.25	2.59
I10	1.25	4.75	5.88	3.75	4.38	5.50	3.63	4.75	3.25	**6.88**	3.63	4.50	3.75	5.13	2.13	4.75	4.24
I11	1.13	2.13	4.50	2.50	2.38	2.50	3.00	2.13	2.25	**6.50**	2.25	2.38	2.50	2.38	1.63	2.13	2.64
I12	2.13	2.38	4.38	3.63	3.38	3.00	3.88	2.38	2.63	**4.88**	3.63	3.50	3.63	3.63	2.00	2.38	3.21
I13	1.13	3.38	6.38	5.00	4.75	4.00	5.13	3.38	4.00	**6.75**	4.50	5.13	5.00	4.50	1.63	3.38	4.25
I14	2.13	1.00	2.88	2.25	2.25	1.50	1.88	1.00	2.00	**3.00**	2.75	2.13	2.25	2.75	1.75	1.00	2.03
I15	3.38	6.13	**8.63**	7.13	6.88	5.63	5.75	6.13	5.13	8.50	7.00	7.38	7.13	7.13	1.63	6.13	6.23
I16	3.63	6.25	5.63	3.75	3.88	5.38	4.00	**6.25**	3.75	5.88	3.38	4.25	3.75	3.38	4.13	6.25	4.59
I17	5.00	9.50	9.88	9.63	9.13	9.88	10.13	9.50	10.00	**10.88**	10.00	9.50	9.63	9.63	5.50	9.50	9.20
Mean	4.53	9.26	11.38	9.65	9.81	9.64	9.60	9.26	8.50	**11.79**	9.82	10.04	9.65	10.01	3.34	9.26	9.10

There is a degree of variation in the performance of the 16 similarity coefficients (*N* objects) by each of the 17 activity classes (*k* judges). The ranks in Tables 5 and 6 are assigned according to Tables 3 and 4, respectively. The degree of agreement between the rankings assigned can be determined by a statistical analysis called the “Kendall Coefficient of Concordance” [37]. This can be calculated by Eq (14).

$$\begin{array}{c} W=\frac{12\sum {\bar{R}}_{i}^{2}-3N{\left(N+1\right)}^{2}}{N\left(N^{2}-1\right)-\frac{\sum T_{j}}{k}}, \end{array}$$ (14)

where ${\bar{R}}_{i}$ is the average of the ranks assigned to the *i*-th object. *T*_*j*_ is a correction factor calculated by Eq (15).

$$\begin{array}{c} T_{j}=\sum_{i=1}^{g_{j}}\left(t_{i}^{3}-t_{i}\right) \end{array}$$ (15)

where *t*_*i*_ is the number of tied ranks in the *i*-th grouping of ties, and *g*_*i*_ is the number of groups of ties in the *j*-th rank. The significance of the computed value of *W* can be obtained from the table of critical values for *N* ≤ 7 [37] or from a table of the chi-square distribution with *N* − 1 degrees of freedom for *N* > 7. We can calculate chi-square from

$$\begin{array}{c} k\left(N-1\right)W∼\chi_{N-1}^{2} \end{array}$$ (16)

The computed values of *W* for similarity searching and WS-ELM are 0.8103, and 0.5161, respectively, which correspond to χ^2^ values of 206.64, and 131.61, respectively (*p* < 0.001 for 15 degrees of freedom). Because agreement between various rankings of the same set of activity classes is significant, this leads to the following orderings in the similarity searching case:

$$\begin{array}{c} C10>C03>C12>C05>C14>C04≃C13>C11>\\ \;C07>C01>C09>C15>C02≃C08≃C16>C06 \end{array}$$

The rank of the 16 coefficients in WS-ELM case is as follows:

$$\begin{array}{c} C10>C03>C12>C14≃C07>C05≃C11>C04≃>\\ \;C13>C06>C02≃C08≃C16>C09>C15>C01 \end{array}$$

**Table 5:** Ranks assigned to 16 similarity coefficients–similarity searching–by 17 activity classes from Table 3.

Class	Similarity Coefficient															
	C01	C02	C03	C04	C05	C06	C07	C08	C09	C10	C11	C12	C13	C14	C15	C16
I01	9.0	14.0	2.0	6.5	4.5	16.0	11.0	14.0	10.0	1.0	8.0	3.0	6.5	4.5	12.0	14.0
I02	9.0	14.5	2.0	7.5	6.0	14.5	12.0	14.5	10.0	1.0	5.0	4.0	7.5	3.0	11.0	14.5
I03	11.0	14.5	2.0	6.5	5.0	14.5	3.0	14.5	10.0	1.0	9.0	4.0	6.5	8.0	12.0	14.5
I04	9.0	15.0	2.0	7.5	6.0	13.0	11.0	15.0	10.0	1.0	4.0	5.0	7.5	3.0	12.0	15.0
I05	9.0	13.0	2.0	7.0	7.0	15.0	16.0	13.0	10.0	1.0	3.5	3.5	7.0	5.0	11.0	13.0
I06	14.0	11.0	3.0	7.0	4.5	13.0	1.0	11.0	15.5	2.0	9.0	4.5	7.0	7.0	15.5	11.0
I07	10.0	14.5	2.0	4.5	7.5	14.5	9.0	14.5	11.0	1.0	4.5	7.5	4.5	4.5	12.0	14.5
I08	11.0	14.5	2.0	6.0	6.0	14.5	3.0	14.5	10.0	1.0	9.0	4.0	6.0	8.0	12.0	14.5
I09	11.0	14.5	2.0	6.0	4.0	14.5	3.0	14.5	10.0	1.0	9.0	6.0	6.0	8.0	12.0	14.5
I10	15.0	12.5	3.0	9.5	7.5	12.5	2.0	12.5	5.0	1.0	7.5	5.0	9.5	5.0	16.0	12.5
I11	4.0	14.0	2.0	6.5	6.5	16.0	12.0	14.0	11.0	1.0	9.5	6.5	6.5	9.5	3.0	14.0
I12	6.0	14.5	2.0	6.0	6.0	14.5	12.0	14.5	10.0	1.0	6.0	6.0	6.0	6.0	11.0	14.5
I13	11.0	13.5	2.0	8.0	5.0	13.5	3.0	13.5	5.0	1.0	10.0	5.0	8.0	8.0	16.0	13.5
I14	4.5	14.5	1.5	4.5	9.0	14.5	12.0	14.5	10.0	1.5	4.5	7.5	4.5	7.5	11.0	14.5
I15	9.0	14.5	2.0	7.0	7.0	14.5	12.0	14.5	11.0	1.0	3.5	5.0	7.0	3.5	10.0	14.5
I16	11.0	14.5	3.0	5.0	5.0	14.5	2.0	14.5	10.0	1.0	9.0	7.0	5.0	8.0	12.0	14.5
I17	11.0	14.5	1.5	7.0	3.5	14.5	9.0	14.5	10.0	1.5	7.0	3.5	7.0	5.0	12.0	14.5
Mean	9.7	14.0	2.1	6.6	5.9	14.4	7.8	14.0	9.9	1.1	6.9	5.1	6.6	6.1	11.8	14.0

**Table 6:** Ranks assigned to 16 similarity coefficients–ELM–by 17 activity classes from Table 4.

Class	Similarity Coefficient															
	C01	C02	C03	C04	C05	C06	C07	C08	C09	C10	C11	C12	C13	C14	C15	C16
I01	15.0	11.0	6.0	4.5	2.0	7.0	13.0	11.0	14.0	3.0	8.0	1.0	4.5	9.0	16.0	11.0
I02	15.0	11.0	9.0	3.5	6.0	8.0	14.0	11.0	13.0	7.0	2.0	5.0	3.5	1.0	16.0	11.0
I03	15.0	10.0	2.0	12.5	8.0	7.0	6.0	10.0	14.0	1.0	4.0	5.0	12.5	3.0	16.0	10.0
I04	15.0	9.5	1.0	13.5	7.0	12.0	4.0	9.5	9.5	2.0	6.0	5.0	13.5	3.0	16.0	9.5
I05	15.0	12.0	2.0	7.5	4.0	9.0	4.0	12.0	14.0	1.0	6.0	4.0	7.5	10.0	16.0	12.0
I06	14.5	5.0	8.0	12.5	10.0	2.0	1.0	5.0	10.0	3.0	14.5	10.0	12.5	16.0	7.0	5.0
I07	16.0	11.0	1.0	5.0	3.0	13.5	9.0	11.0	13.5	2.0	8.0	5.0	5.0	7.0	15.0	11.0
I08	15.0	13.0	1.0	9.5	6.5	11.0	5.0	13.0	6.5	2.0	8.0	3.0	9.5	4.0	16.0	13.0
I09	13.0	4.5	2.0	11.5	14.5	4.5	7.5	4.5	10.0	1.0	9.0	14.5	11.5	7.5	16.0	4.5
I10	16.0	6.0	2.0	10.5	9.0	3.0	12.5	6.0	14.0	1.0	12.5	8.0	10.5	4.0	15.0	6.0
I11	16.0	13.0	2.0	5.0	8.0	5.0	3.0	13.0	10.5	1.0	10.5	8.0	5.0	8.0	15.0	13.0
I12	15.0	13.0	2.0	5.5	9.0	10.0	3.0	13.0	11.0	1.0	5.5	8.0	5.5	5.5	16.0	13.0
I13	16.0	13.0	2.0	5.5	7.0	10.5	3.5	13.0	10.5	1.0	8.5	3.5	5.5	8.5	15.0	13.0
I14	8.5	15.0	2.0	6.0	6.0	13.0	11.0	15.0	10.0	1.0	3.5	8.5	6.0	3.5	12.0	15.0
I15	15.0	10.0	1.0	5.0	8.0	13.0	12.0	10.0	14.0	2.0	7.0	3.0	5.0	5.0	16.0	10.0
I16	14.0	2.0	5.0	12.0	10.0	6.0	9.0	2.0	12.0	4.0	15.5	7.0	12.0	15.5	8.0	2.0
I17	16.0	11.5	5.5	8.0	14.0	5.5	2.0	11.5	3.5	1.0	3.5	11.5	8.0	8.0	15.0	11.5
Mean	14.7	10.0	3.1	8.1	7.8	8.2	7.0	10.0	11.2	2.0	7.8	6.5	8.1	7.0	14.5	10.0

WS-ELM is then compared with the similarity searching technique. According to Tables 3 and 4, WS-ELM can achieve higher maximum percentage actives retrieved at 9.10% than similarity searching does at 7.19% on average across 17 activity classes, 16 similarity coefficients, four fingerprints, and ten runs. The *t*-test is used to test the significance level of the difference between the means of two independent samples [37]. It is confirmed that WS-ELM can perform better than similarity searching on average at *p* < 0.001.

Next, the performance of Sokal/Sneath(1) on similarity searching and WS-ELM is analysed. As shown in Tables 3 and 4, similarity searching and WS-ELM can achieve 11.79% and 12.16% of maximum percentage actives retrieved, respectively. However, it is inconclusive that similarity searching with Sokal/Sneath(1) is outperforming WS-ELM with Sokal/Sneath(1) at *p* = 0.5759.

**Fig 2:**
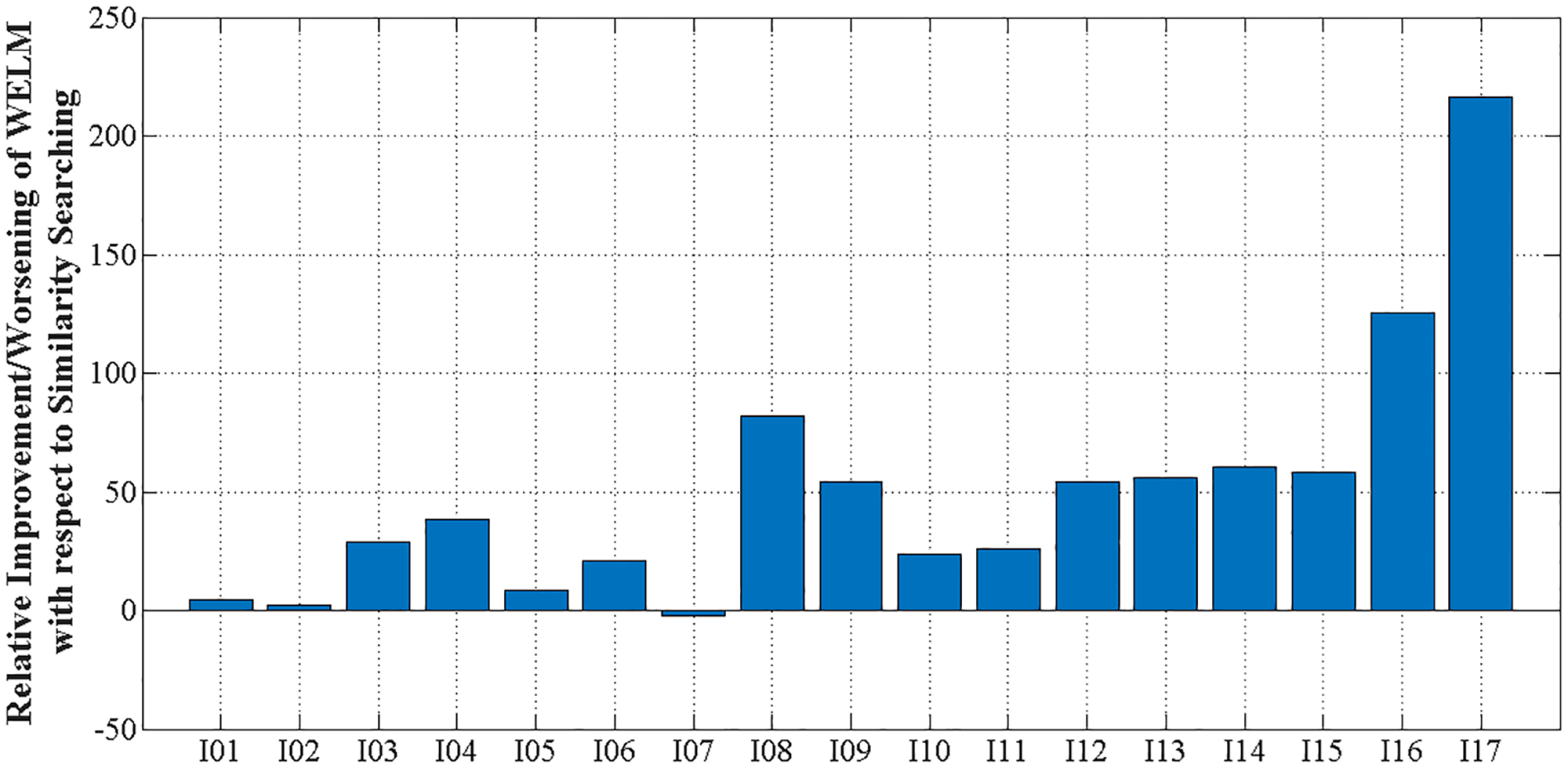
Relative improvement/worsening with respect to similarity searching for top 1% retrieved–average across ten runs, 16 similarity coefficients, and four fingerprints.

Fig 2 shows relative improvement or worsening of WS-ELM with respect to similarity searching on average across 16 similarity coefficients, four fingerprints, and 10 runs. The entries are sorted by MPS score. It is hardly surprising that WS-ELM performs better than similarity searching. This is because similarity searching only uses active molecules in its training set while WS-ELM has a proper training set consisting of active and inactive molecules. WS-ELM was more effective than similarity searching in 16 out of 17 cases, especially in the cases with low MPS (heterogeneous). This means that including inactive molecules in the training sets can improve overall performance. However, it might not be very useful in some homogeneous classes, i.e. I01, I02, I5, and I07.

**Fig 3:**
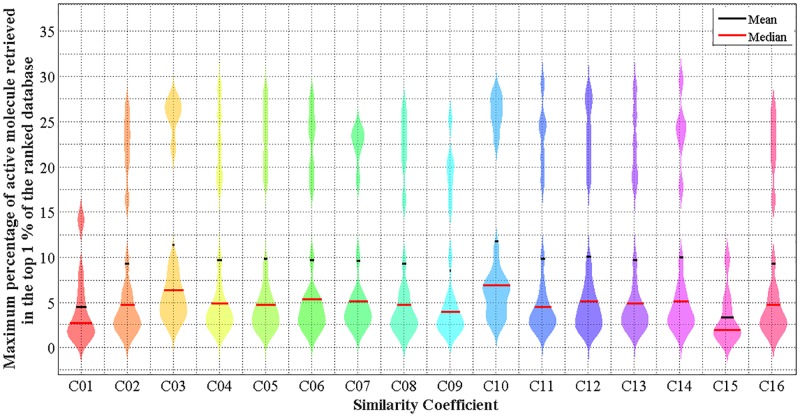
Violin plot of maximum percentage of active molecules retrieved in the top 1% with WS-ELM in conjunction with 16 different similarity coefficients–averaged across ten runs, 17 activity classes, and four fingerprints.

**Fig 4:**
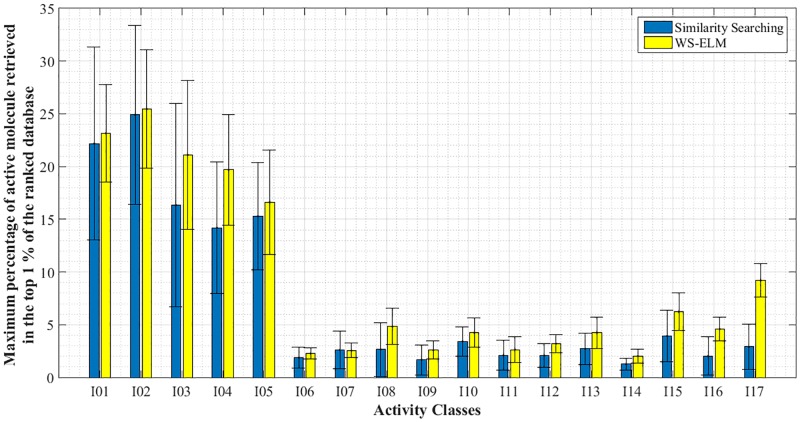
Maximum percentage of active molecules retrieved in the top 1% with WS-ELM and similarity searching in 17 activity classes–averaged across ten runs, 16 similarity coefficients, and four fingerprints.

Further analysis is conducted by using a violin plot (as shown in Fig 3) to evaluate the distribution of the results for WS-ELM in conjunction with each similarity coefficient. It is clearly seen that there are two distinct distributions in each coefficient. These two distributions reflect those activity classes with high and low MPS scores. The distribution with higher performance contains activity I1, I2, I3, I4, and I5 with average MPS of 0.21 while distributions with lower hit rate contain the remaining activity classes with average MPS of 0.18. In other words, the five most homogeneous activity classes in the MUV dataset could achieve higher hit rates compared to the others. On the other hand, if the active molecules are very structurally heterogeneous, it is difficult to achieve a high hit rate in that activity class as shown in Fig 4.

**Fig 5:**
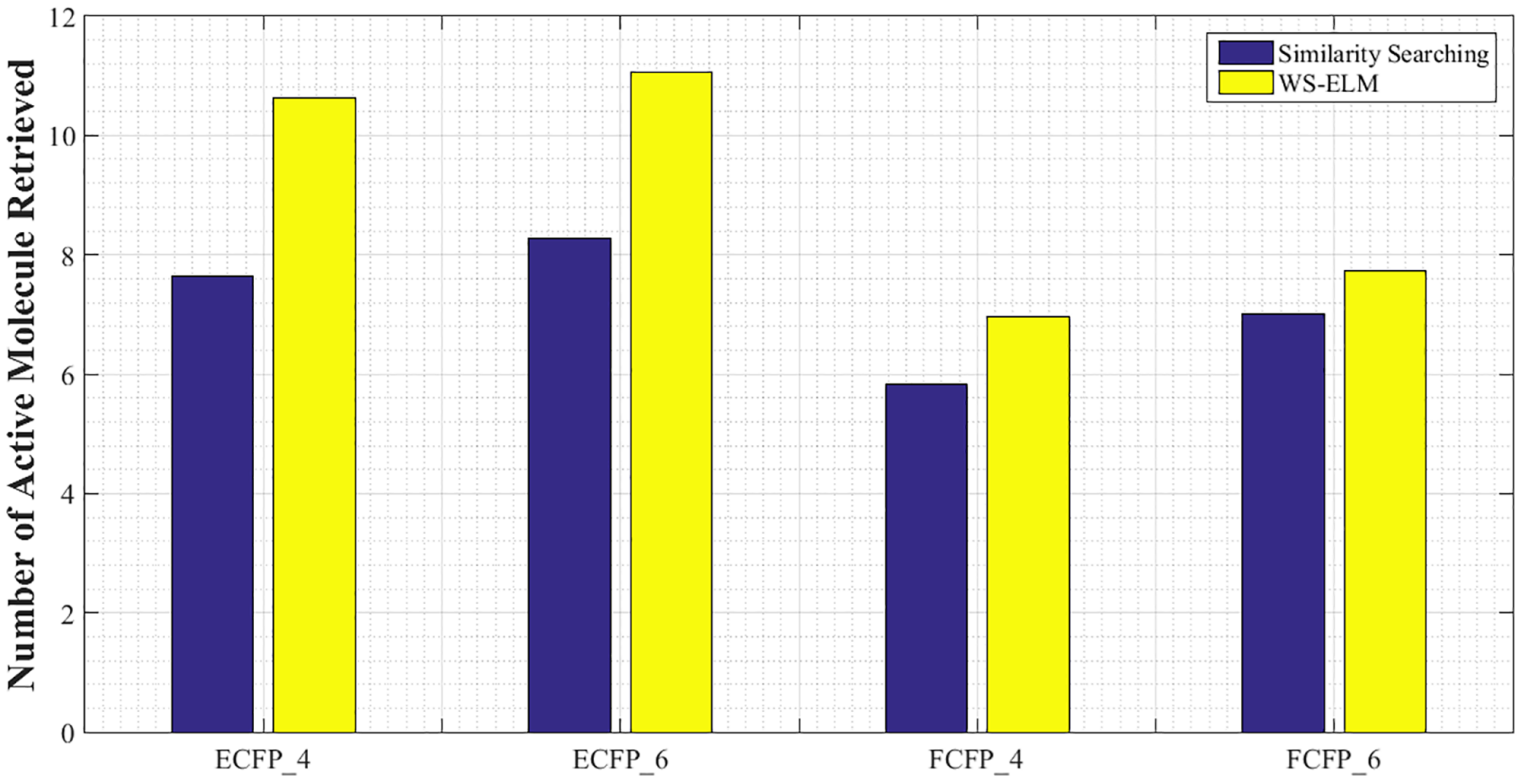
Maximum percentage of active molecules retrieved with WS-ELM and similarity searching using four different fingerprints–averaged across 17 activity classes, 16 similarity coefficients, and 10 runs.

Fig 5 shows maximum percentage of actives retrieved with WS-ELM and similarity searching using different fingerprints–averaged across all activity classes and all similarity coefficients. Representing molecules with ECFP_6 fingerprint enables retrieving the most actives on average in both WS-ELM and similarity searching. FCFP_4 fingerprint performs worst on average. Again, Kendall Coefficient of Concordance is applied in order to obtain ordering in four fingerprints–4 objects–and 17 judges. The computed *W* values are 0.1657 and 0.1352 for WS-ELM and similarity searching, respectively. According to these values, the chi-square values yield 84.5153 and 68.9718, respectively; both are significant at the 0.001 level of statistical significance. This suggests the same orderings in fingerprint case for both WS-ELM and similarity searching:

$$\begin{array}{c} \text{ECFP}\_6>\text{ECFP}\_4>\text{FCFP}\_6>\text{FCFP}\_4. \end{array}$$

### A comparison of CWS-ELM and WS-ELM with the best two similarity coefficients

The two best similarity coefficients–Sokal/Sneath(1) and Jaccard/Tanimoto–for MUV dataset from the first part are employed in the proposed CWS-ELM algorithm. The proposed algorithms are compared with WS-ELM on the same framework. Maximum percentage of active molecules retrieved in the top 1% and number of hidden nodes used in the model are reported in Table 7. Each element is an average across four fingerprints and 10 runs. The proposed algorithm is reported as CWS-ELM_KMC_ and CWS-ELM_SVC_ for CWS-ELM in conjunction with *k*-means clustering and SVC, respectively.

**Table 7:** The percentage hit rate in the top 1% of the ranked database retrieved by WS-ELM and CWS-ELM in conjunction with Jaccard/Tanimoto (JT) and Sokal/Sneath(1) (SN1). Figures in bold face represent the best performance.

Class	WS-ELM		CWS-ELM			
	Random		K-Means		SVC	
	JT	SN1	JT	SN1	JT	SN1
I01	25.50	25.88	28.88	28.50	32.88	**33.25**
	54.24	48.91	51.09	53.79	49.72	69.87
I02	27.13	28.00	27.25	25.88	28.50	**29.63**
	54.85	45.51	54.32	46.16	50.21	71.03
I03	27.25	27.63	**28.38**	26.63	24.13	27.50
	57.59	57.26	62.97	63.13	54.79	74.82
I04	26.00	25.25	20.38	22.88	25.25	**30.88**
	52.56	58.63	47.24	55.29	55.56	79.31
I05	22.13	23.00	**24.63**	23.50	21.13	23.00
	63.01	68.76	69.84	71.63	52.32	73.99
I06	2.25	2.63	2.25	2.25	**4.13**	3.88
	37.26	36.51	34.62	37.40	52.21	66.28
I07	3.75	3.38	3.00	2.75	3.63	**4.00**
	52.22	55.82	49.81	50.19	53.32	74.41
I08	7.63	7.25	7.00	8.38	7.25	**8.88**
	52.94	54.00	51.47	47.78	53.65	68.66
I09	3.75	4.25	3.88	4.25	4.38	**5.25**
	42.60	46.38	39.71	37.76	52.71	67.00
I10	5.88	6.88	6.50	5.75	6.88	**8.50**
	55.90	64.40	59.72	62.51	51.26	71.51
I11	4.50	**6.50**	5.13	6.38	6.13	6.00
	50.57	55.26	50.28	54.00	51.32	69.66
I12	4.38	**4.88**	4.75	**4.88**	4.75	**4.88**
	51.84	55.53	51.00	50.51	51.00	69.50
I13	6.38	6.75	6.63	6.63	**6.88**	6.75
	51.84	56.22	48.25	47.44	52.12	71.26
I14	2.88	**3.00**	1.63	1.25	1.88	1.75
	36.93	41.22	43.65	49.06	51.90	67.59
I15	8.63	8.50	8.25	8.13	**9.13**	8.25
	62.90	67.49	67.78	69.82	54.74	71.01
I16	5.63	5.88	4.63	4.75	5.63	**7.50**
	46.60	46.37	38.59	41.44	53.76	69.51
I17	9.88	10.88	10.00	11.00	**12.00**	11.50
	60.29	66.09	56.71	59.06	52.68	71.53
Mean	11.38	11.79	11.36	11.40	12.03	**13.02**
	52.01	54.38	51.59	52.76	52.54	71.00

In the overall picture, the proposed CWS-ELM yields the highest performance measure in 15/17 activity classes. The best technique is CW-ELM_SVC_ in conjunction with Sokal/Sneath(1) which achieves the best percentage of active molecules retrieved in 9/17 cases at 13.02% on average across all activity classes, but it requires the highest number of nodes in the hidden layer at 71.0%. This is followed by CW-ELM_SVC_ in conjunction with Jaccard/Tanimoto which achieved a 12.03% hit rate and exhibited high accuracy in 4/17 cases. However, CW-ELM_KMC_’s performance is slightly worse than WS-ELM because it contains a smaller number of hidden nodes on average than WS-ELM. The correlation coefficient between the mean percentage of hit rates and number of nodes used in the model is 0.93 which is considered very highly correlated. Due to the high degree of diversity in the dataset, therefore, the number of nodes in the hidden layer can directly affect the performance of the model. If the model is too simple, it can degrade the performance of the classifier.

As our proposed algorithm embeds two clustering techniques to select the represented samples in WS-ELM–one selects the centroids of the clusters and the other utilises support vectors bounding the clusters, they are different in nature. Considering the same number of clusters in the space, SVC requires more than one support vector to bound and identify the cluster while *k*-means clustering needs only one centroid to represent the cluster. Therefore, there is a high chance of SVC performing better than *k*-means clustering in this dataset as they are very diverse.

Again, we applied the Kendall Coefficient of Concordance to test the significance on the ranking of six contenders in Table 8. The computed *W* is 0.2581–leading to a χ^2^ of 21.94–which indicates that the results are highly statistically significant. This gives the following ranking:

$$\begin{array}{c} {\text{CWS-ELM}}_{\text{SVC-SN}}>{\text{WS-ELM}}_{\text{SN}}>{\text{CWS-ELM}}_{\text{SVC-JT}}>\\ \;{\text{CWS-ELM}}_{\text{KMC-SN}}>{\text{CWS-ELM}}_{\text{KMC-JT}}≃{\text{WS-ELM}}_{\text{JT}} \end{array}$$

It is clear that CWS-ELM_SVC_ is the best contender among all while the worst is WS-ELM_JT_.

**Table 8:** Ranks assigned to the performances of 6 classifiers by 17 activity classes from Table 7.

Class	WS-ELM		CWS-ELM			
	Random		K-Means		SVC	
	JT	SN1	JT	SN1	JT	SN1
I01	6.00	5.00	3.00	4.00	2.00	1.00
I02	5.00	3.00	4.00	6.00	2.00	1.00
I03	4.00	2.00	1.00	5.00	6.00	3.00
I04	2.00	3.50	6.00	5.00	3.50	1.00
I05	5.00	3.50	1.00	2.00	6.00	3.50
I06	5.00	3.00	5.00	5.00	1.00	2.00
I07	2.00	4.00	5.00	6.00	3.00	1.00
I08	3.00	4.00	6.00	2.00	5.00	1.00
I09	6.00	3.50	5.00	3.50	2.00	1.00
I10	5.00	2.50	4.00	6.00	2.50	1.00
I11	6.00	1.00	5.00	2.00	3.00	4.00
I12	6.00	2.00	4.50	2.00	4.50	2.00
I13	6.00	2.50	4.50	4.50	1.00	2.50
I14	2.00	1.00	5.00	6.00	3.00	4.00
I15	2.00	3.00	4.50	6.00	1.00	4.50
I16	3.50	2.00	6.00	5.00	3.50	1.00
I17	6.00	4.00	5.00	3.00	1.00	2.00
Mean	4.38	2.91	4.38	4.29	2.94	2.09

**Fig 6:**
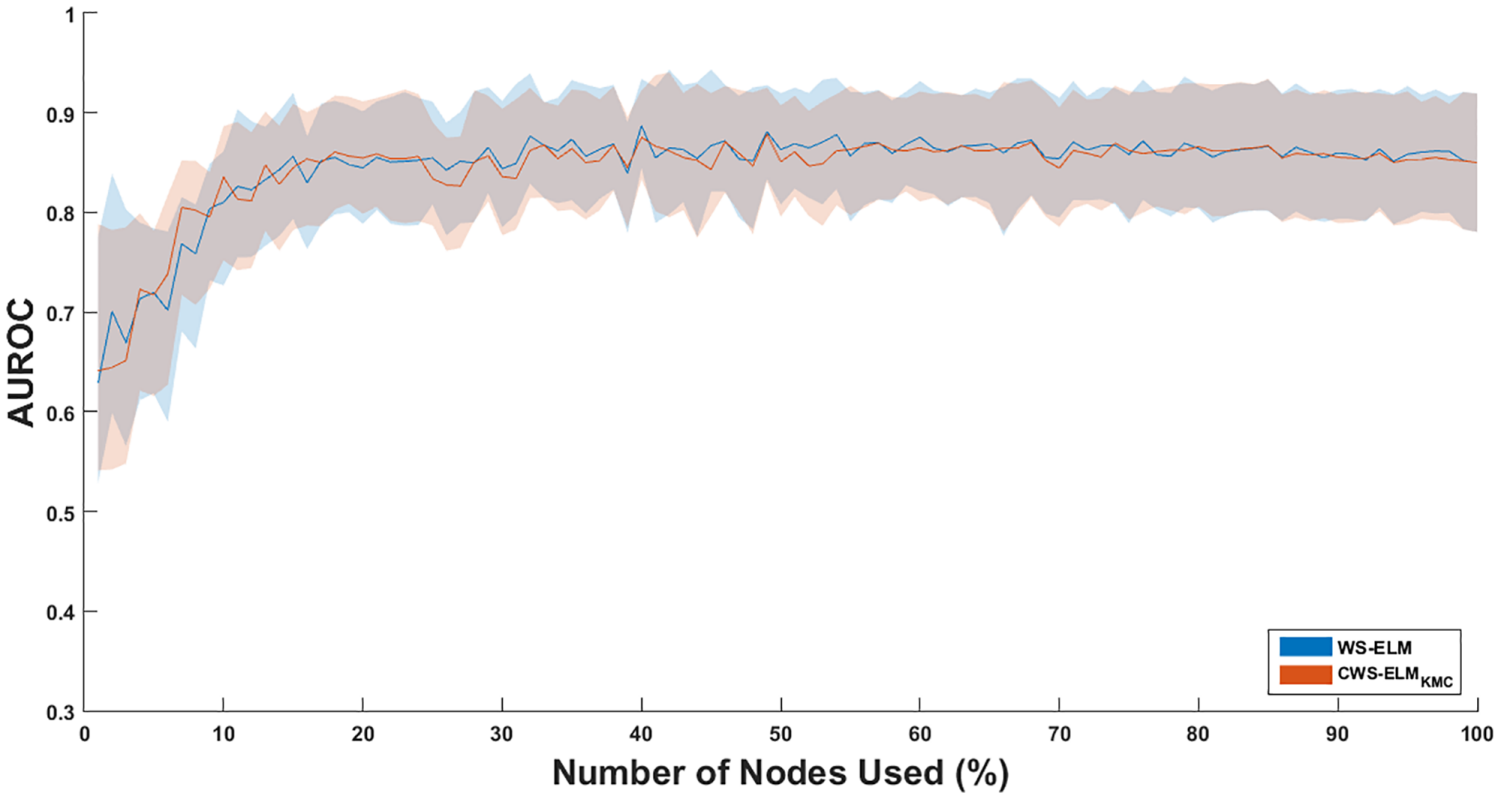
Effect of AUROC when the number of hidden nodes in WS-ELM and CWS-ELM_KMC_ is changed in activity class I01. Solid lines represent mean values while shaded areas represent error/confidence bounds. The upper and lower bounds of each node are based on the standard deviation.

**Fig 7:**
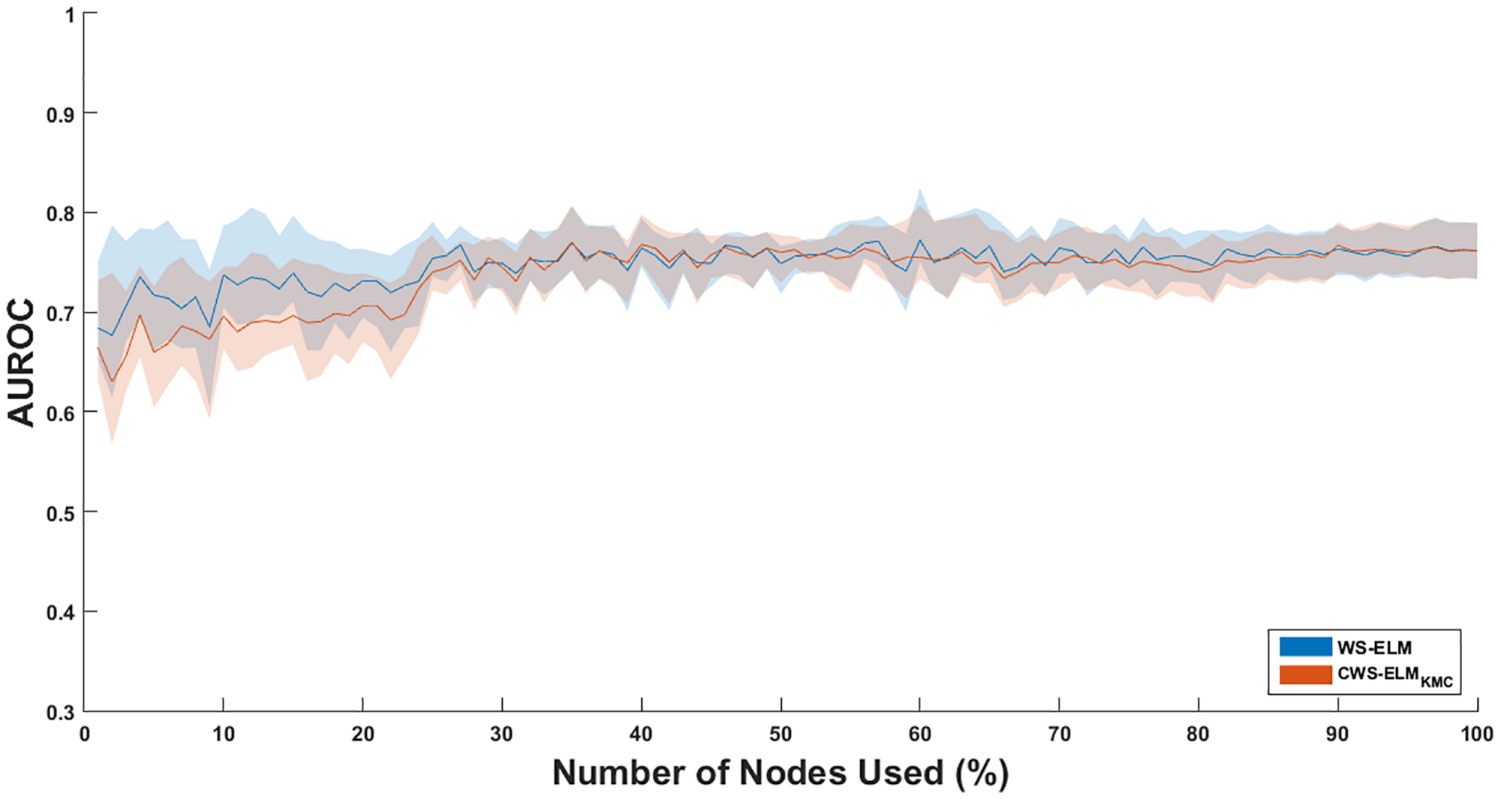
Effect of AUROC when the number of hidden nodes in WS-ELM and CWS-ELM_KMC_ is changed in activity class I17. Solid lines represent mean values while shaded areas represent error/confidence bounds. The upper and lower bounds of each node are based on the standard deviation.

Furthermore, the effect of the number of nodes in the hidden layer on the performance is investigated. The most homogeneous (I1) and the most diverse (I17) classes in the dataset with ECFP_6 fingerprint are evaluated. The regularisation parameter for each model–with a different number of nodes used–is tuned by five-fold cross validation on the basis of AUROC. Again, the experiment is conducted ten times with different random splits. In this experiment, only WS-ELM and CWS-ELM_KMC_ are evaluated because the number of hidden nodes of these two can be directly adjusted and compared. Unlike the CWS-ELM_SVC_, the number of nodes depends on *C*_*s*_. The results of I01 and I17 are displayed in Figs 6 and 7, respectively.

It is clear that CWS-ELM_KMC_ is better than WS-ELM in number of actives retrieved when a small number of nodes is used (1–28%) in the model for I17 as shown in Fig 7. Moreover, it is more robust than WS-ELM resulting in smaller standard deviations in the performances. This means that carefully selected samples in the hidden node is important. According to Fig 6, CWS-ELM_KMC_ is comparable to WS-ELM in I01. Comparing performances of the classifiers in both activity classes, AUROC of I01 achieves convergence at 15% of number of nodes used while the convergence of AUROC in I17 occurs at 30% of number of nodes used. This shows that the performance of classifiers on I01 can achieve convergence quicker than I17.

**Fig 8:**
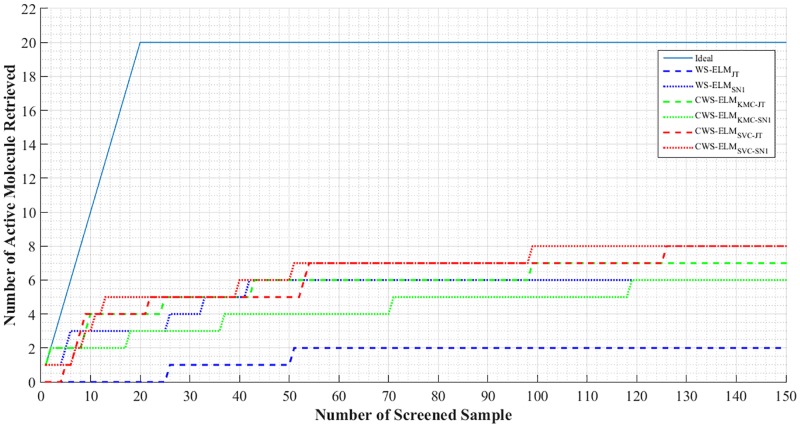
Enrichment plot for the top 1% of the sorted library for each performer with ECFP_6 fingerprint on activity class I01.

**Fig 9:**
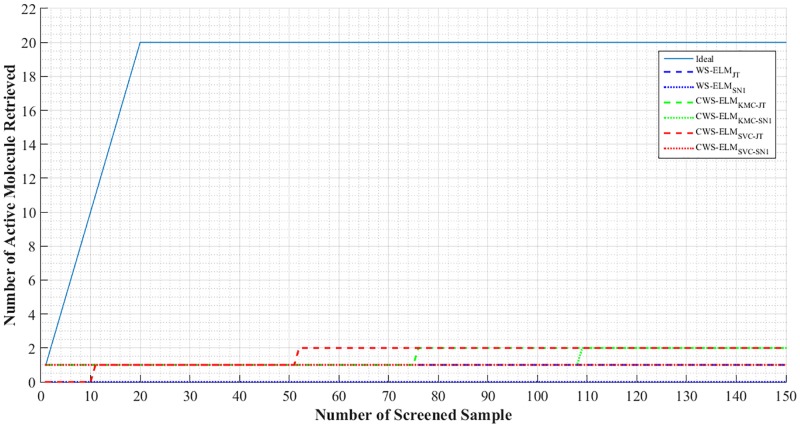
Enrichment plot for the top 1% of the sorted library for each performer with ECFP_6 fingerprint on activity class I17.

We also show an enrichment plot which is a very useful method for evaluating the quality of virtual screening methods. It is a cumulative sum plot of the active molecules retrieved from the top 1% of the ranked database. Figs 8 and 9 show enrichment plots for I01 and I17, respectively. Clearly, CWS-ELM’s performances are better than the conventional WS-ELM_JT_ in both I01 and I17. Performances by all methods on I01, the most homogeneous activity class, are better than on I17, the most heterogeneous activity class.

**Fig 10:**
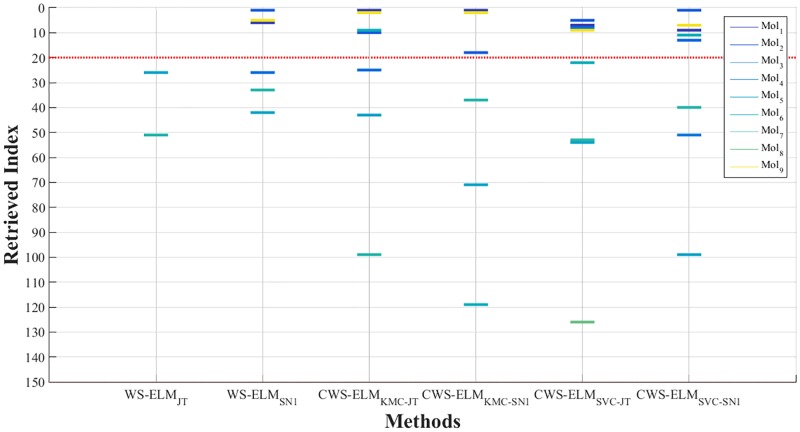
Molecules retrieved by different methods in top 1% of the ranked database for activity class I01.

**Fig 11:**
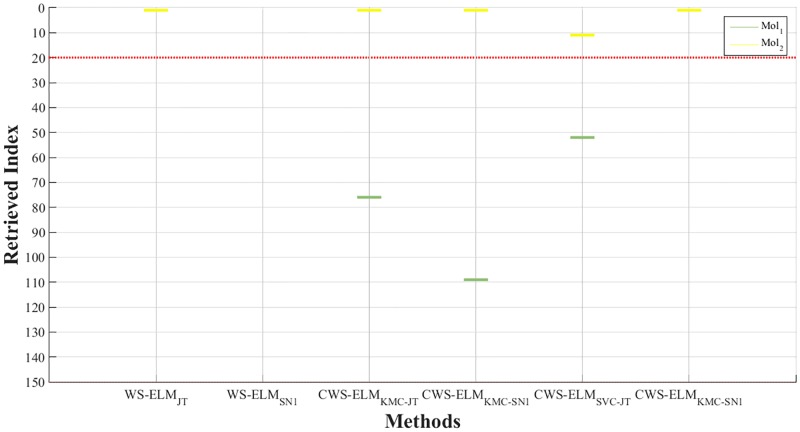
Molecules retrieved by different methods in top 1% of the ranked database for activity class I17.

In addition to comparing the overall performance results by using enrichment plots, the individual molecules that are being retrieved are shown in Figs 10 and 11 for activity I01 and I17, respectively. It can be seen that CWS-ELM_SVC-SN_ is the best in I01. Basically, any molecules retrieved by other approaches can be retrieved in the top 1% of the list by CWS-ELM_SVC-SN_ but in different orders. This is because Sokal/Sneath(1) is a modified version of the Jaccard/Tanimoto function as mentioned in the previous section. In I17 case, WS-ELM_SN_ fails to retrieve any active molecules in the top 1% while the other methods can retrieve one or two active molecules.

### A comparison of CWS-ELM and WS-ELM with the best similarity coefficient against other approaches

The proposed methods CWS-ELM and WS-ELM are compared against other approaches, namely SVM, RF, and Similarity Searching. Apart from RF, all other methods are based on Sokal/Sneath(1) coefficient. The hyper-parameters of SVM and RF are tuned with the same framework as the proposed methods. As mentioned earlier, there are many criteria to evaluate the algorithms but, in the previous experiments, AUROC is chosen for its simplicity, and the percentage hit rate in the top 1% which gives the same picture as EF. However, AUROC has been criticised because it is a global measure that does not pay attention to the top-ranked molecules, therefore Truchon & Bayly proposed a generalised ROC metric called “Boltzmann-Enhanced Discrimination of ROC” (BEDROC) which considers the early recognition problem [34].

**Fig 12:**
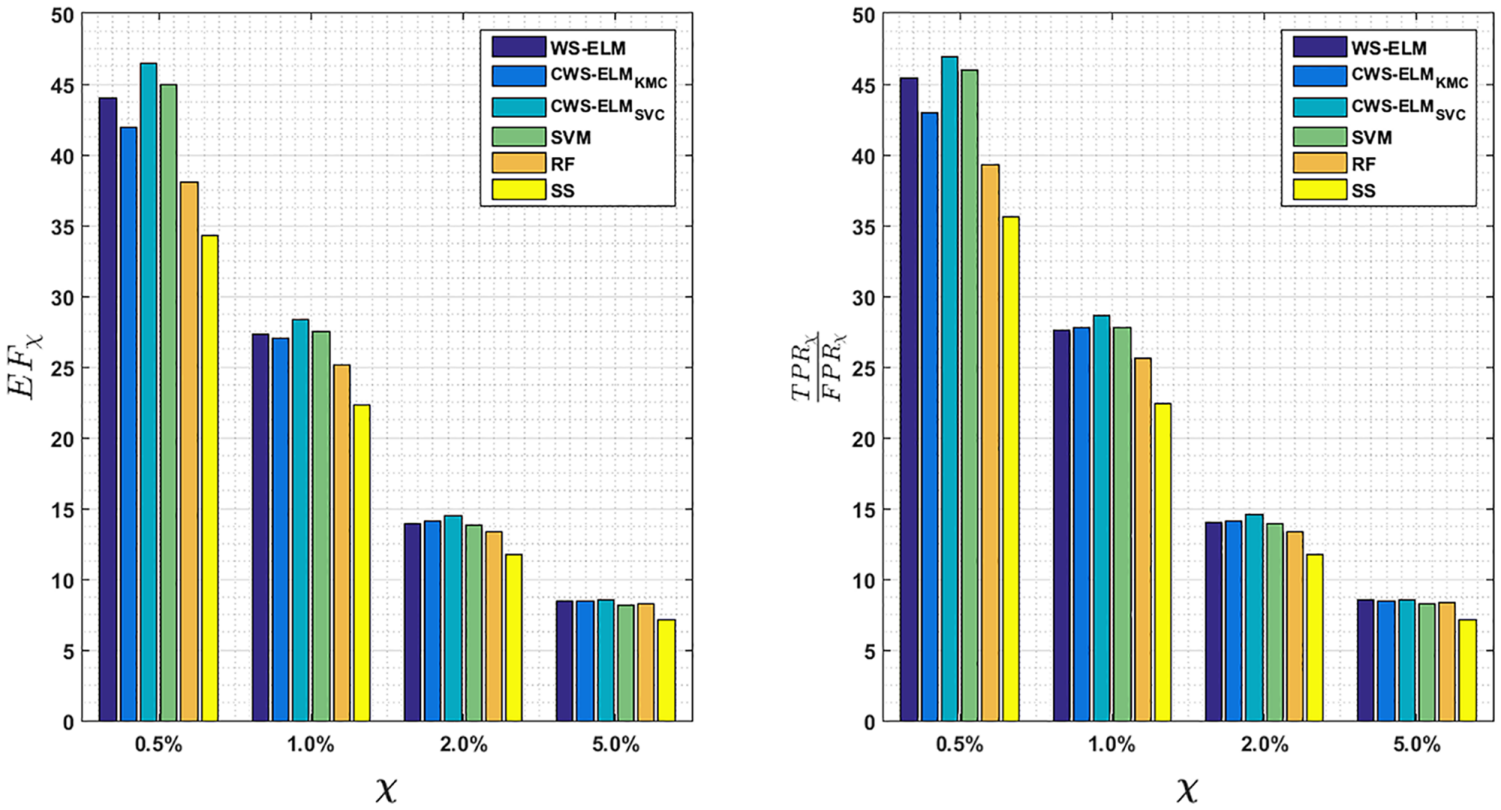
Early recognition criteria suggested by [35, 38]. (Left) EF (Right) Ratio of true positive rate to the false positive rate, at 0.5%, 1.0%, 2.0%, and 5.0% of the ranked database for WS-ELM and its variants, SVM, RF, and Similarity Searching (SS). Each bar represents the mean value across all activity classes and ten runs.

However, the best approaches to evaluate the virtual screening task are recommended [35, 38], and EF gives very much the same results as BEDROC but is easier to understand [36]. Therefore we follow the evaluations suggested in [35] by reporting the following measures: (i) EF at 0.5%, 1.0%, 2.0%, and 5.0%, and (ii) The ratio of true positive to false positive rates at 0.5%, 1.0%, 2.0%, and 5.0%. Fig 12 shows EF and the ratio of true positive to false positive rates at the top 0.5%, 1.0%, 2.0%, and 5.0% of the ranked database. Both criteria display the same overall picture. CWS-ELM_SVC_ is still the best contender among all other algorithms followed by SVM at EF_0.5%_ and EF_1.0%_. The worst is similarity search technique as expected. These are confirmed by Kendall Coefficient of Concordance (with *N* = 6 and *k* = 17)–*W* values are 0.2186 (*p* < 0.01) and 0.1576 (*p* < 0.05) for EF at 0.5% and 1.0%, respectively–and lead to the following rankings.

$$\begin{array}{l} \;{\text{EF}}_{0.5\;\%}:\\ {\text{CWS-ELM}}_{\text{SVC}}>\text{SVM}>\text{WS-ELM}>{\text{CWS-ELM}}_{\text{KMC}}>\text{RF}>\text{Similarity}\;\text{Searching}\\ \;{\text{EF}}_{1.0\;\%}:\\ {\text{CWS-ELM}}_{\text{SVC}}>\text{SVM}>\text{RF}>\text{WS-ELM}>{\text{CWS-ELM}}_{\text{KMC}}>\text{Similarity}\;\text{Searching} \end{array}$$

Unfortunately, values of *W* are not significant at *p* = 0.05 level in the case of EF of 2.0% and 5.0%.

**Fig 13:**
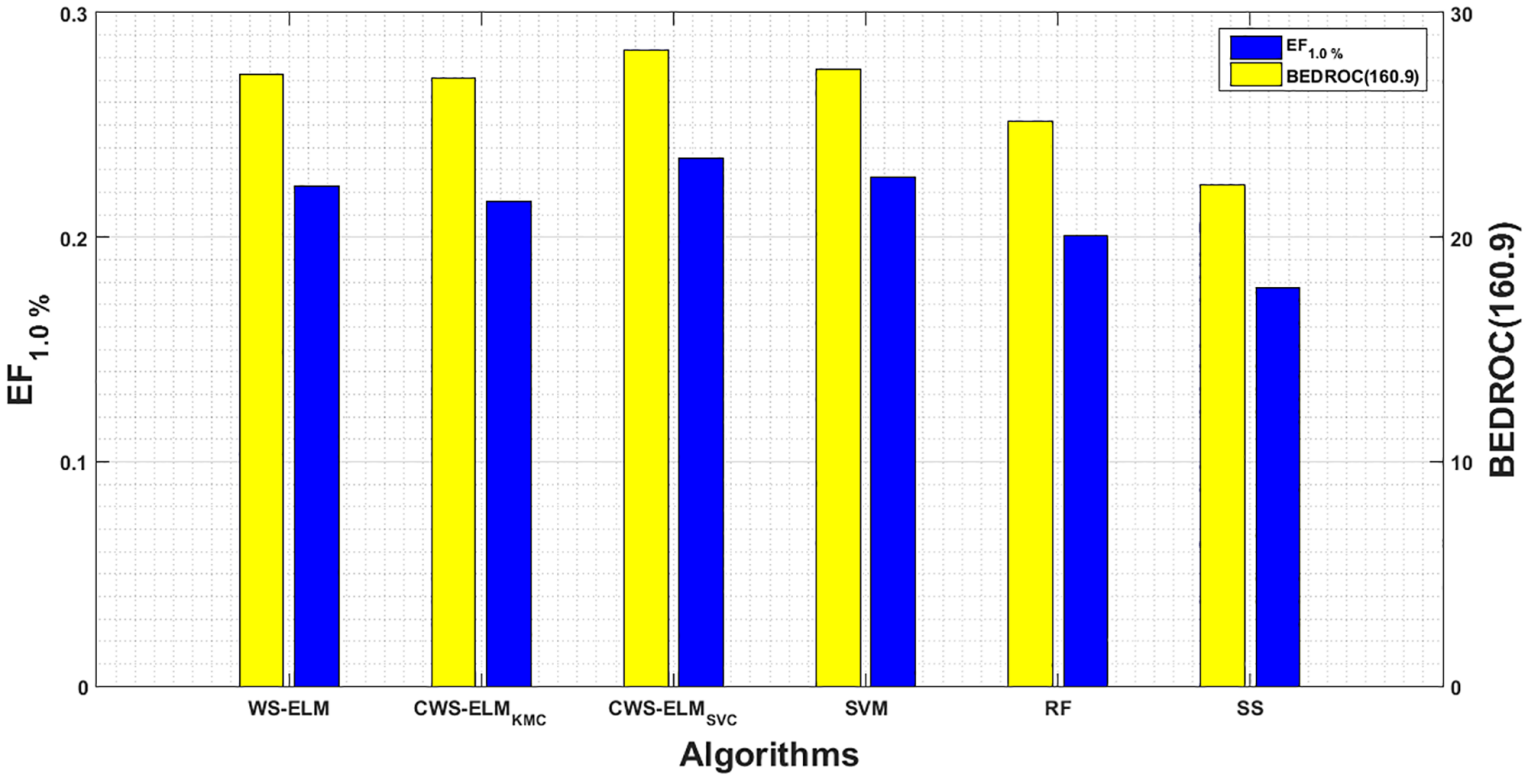
Bar charts showing mean EF and BEDROC at 1.0% of the ranked database for WS-ELM and its variants, SVM, RF, and Similarity Seaching (SS). According to Truchon & Bayly, the top 1% of the ranked database is equivalent to *α* = 160.9 of BEDROC [34].

Furthermore, we also evaluate the task with BEDROC and a parameter *α* which relates to the number of considered top ranked molecules in the database. The higher the value of *α* is, the smaller the considered number of molecules is. As we are interested in the top 1% of the ranked database, *α* is equal to 160.9 (refer to [34]). The BEDROC results are shown in Fig 13 with EF_1.0%_. Again, testing the results with Kendall Coefficient of Concordance (*W* = 0.1585) gives the following ranking at *p* = 0.05:

$${\text{CWS-ELM}}_{\text{SVC}}>\text{SVM}>\text{WS-ELM}>\text{RF}>{\text{CWS-ELM}}_{\text{KMC}}>\text{Similarity}\;\text{Searching}.$$

Moreover, Fig 13 also shows that EF_1.0%_ correlates with BEDROC(160.9) with correlation coefficient of 0.9917. Although EF and BEDROC are strongly correlated, EF does not take into account the ratio of active and inactive molecules while BEDROC does.

## Conclusion

This study proposes a modified ELM, termed WS-ELM, which improves the overall performance of virtual screening tasks. It demonstrates the capability of WS-ELM on the MUV dataset which is known as one of the most challenging datasets. The results show that Sokal/Sneath(1) and Jaccard/Tanimoto are the two best performers in this task among 16 similarity coefficients. Moreover, statistical analysis shows that using the ECFP fingerprint is better than the FCFP fingerprint, and utilising a circular substructure of six diameter bonds is generally better than four diameter bonds. Because of random generation of the weights in hidden nodes, it is not able to guarantee the stability and robustness of WS-ELM. This can lead to a lack of accurate prediction. Thus, WS-ELM is extended as CWS-ELM which adopts a clustering algorithm to enhance its performance, namely *k*-mean clustering and SVC, to carefully select weights in hidden nodes instead of randomly. Experimental results confirm that CWS-ELM performances are better and more robust than WS-ELM. CWS-ELM_SVC-SN_ is the best approach which is consistently listed in the top ranks compared with its variants and other machine learning techniques.
